# Developmental Dysgraphia as a Reading System and Transfer Problem: A Case Study

**DOI:** 10.3389/fpsyg.2016.00149

**Published:** 2016-02-23

**Authors:** Claire M. Fletcher-Flinn

**Affiliations:** School of Psychology, The University of AucklandAuckland, New Zealand

**Keywords:** dysgraphia, specific spelling disability, dyslexia, induced sublexical relations (ISRs), Knowledge Sources theory

## Abstract

This is a case study of an adolescent who had largely overcome his early difficulty in learning to read, but continued to have severe problems with spelling. He had no visual memory impairment, and his letter–sound knowledge and phonemic awareness were at adult levels. Testing revealed that his difficulties in both reading and spelling only manifested when processing unfamiliar words. He was slow and inaccurate when reading non-words, despite a sublexical system dominated by the use of grapheme–phoneme units. It is suggested that limitations in the processing of the reading system were responsible for the lack of an extensive set of induced position-sensitive sublexical representations (ISRs) that are contextually dependent. This would have serious consequences for transfer to spelling.

## Introduction

Reading and writing are important skills learned during childhood. It is estimated that up to 10% of children have difficulty learning to read (Snowling, 2013), and much research effort has focused on understanding the cognitive deficits associated with developmental dyslexia. There has been far less emphasis on spelling and writing, despite being at least as severe, if not worse, than the reading difficulties experienced by dyslexics (Ellis, 1993), with poor spelling often persisting into adulthood among compensated dyslexics (Critchley and Critchley, 1978; Miles, 1983; Bruck, 1990). The purpose of this study was to examine a case of developmental dysgraphia from a learning perspective, using Knowledge Sources theory (Thompson et al., 1996; Thompson and Fletcher-Flinn, 2006) as an explanatory framework. Although formal generalization from case studies is not warranted, well-chosen cases are useful for tests of falsification (Flyvbjerg, 2006), and for restricting the range of theory application (Fletcher-Flinn, 2014).

### Dysgraphia

Dysgraphia was originally included in the description of dyslexia (Deuel, 1995) but is now differentiated as a component within the broad spectrum of writing disorders, referring specifically to spelling, and illegible handwriting (Adi-Japha et al., 2007). Different strands of theoretical research and proposed causal mechanisms for these two aspects of dysgraphia has separated the field of enquiry further, and is responsible for some confusion in the use of the term (Nicolson and Fawcett, 2011). Cognitive developmental researchers tend to use the term dysgraphia to apply only to the cognitive aspects of spelling (e.g., Frith, 1980), while those interested in writing difficulties (e.g., Deuel, 1995) use the term to refer to motor difficulties in handwriting. This paper focuses on the former.

Much of our knowledge of dysgraphia comes from studies of adults who have suffered brain trauma through accident or stroke. As these adults were literate prior to their injuries, they are considered to have ‘acquired’ the impairments. Various patterns of acquired dysgraphia have been described that parallel the acquired dyslexic categories. Both are based on the dual-route theory (Coltheart, 2005), which separates the reading system into lexical (word) and non-lexical pathways. Familiar words are recognized via the lexical route, and unfamiliar words and non-words are pronounced through a grapheme–phoneme (letter–sound) conversion system for reading and writing. Those individuals with phonological dysgraphia can spell words, but not non-words, and those with surface dysgraphia can spell non-words and regular words but not irregular words. As might be expected, acquired dysgraphia is usually concurrent with acquired dyslexia, and similar errors in reading and writing are reported (Ellis, 1993).

There have been several interesting adult cases, however, in which there is an asymmetry between reading and writing. One case has been reported in which reading was in the normal range but spelling was below expectation. Miceli et al. (1985) reported the case of an Italian adult, FV, with ‘pure dysgraphia’ and no concomitant reading problems. FV had suffered a cardiovascular accident but had recovered to the extent that he could resume his law practice. However, his writing continued to be problematic. On writing to dictation, he made errors on 22% of the words, and 31% of the non-words presented. FV seemed to be aware of his errors, and was able to correct many of them. He also performed well on tests of delayed copying of word and non-words. His difficulty was thought to lie with refreshing the decaying item in the graphemic buffer, which holds the representation of the spelling during the act of writing. It was suggested that FV used the phoneme–grapheme (P–G) conversion system to refresh the graphemic buffer. The P–G conversion system works by transforming the phonological form of the item held in a phoneme buffer to its graphemic form. It was concluded that overreliance on the P–G conversion system instead of a direct lexical representation would result in errors even in a shallow orthography like Italian.

Goulandris and Snowling (1991) described the case of JAS, a university undergraduate student who had severe problems with spelling. She was reported as being a (developmental) surface dyslexic who had largely compensated for her reading difficulties, which was within the normal range on standardized tests. However, further testing showed that she had subtle impairments in reading. She showed a ‘marginal’ regularization effect on low frequency words, and had more difficulty than expected with irregular words, making mainly regularization errors. Her spelling strategies were considered unusual. When presented with words to spell, she “sounded out” individual letters in words before writing them, even when she knew how to spell the item. This idiosyncratic sound based approach to spelling was unreliable, with more errors made on irregular words. Tests of visual processing showed that she had severe deficits on visual memory tasks. It was concluded that these visual memory impairments prevented JAS from establishing detailed orthographic representations in her lexical system, having a severe effect on her spelling.

Both of the adult cases of dysgraphia are similar in that there appeared to be an overreliance on phonology to spell and write words. The question remains as to whether developmental cases of dysgraphia (without reading impairment) have particular difficulty with either establishing precise orthographic representations due to poor visual memory as suggested for JAS, or having access to the representations, as may have been the case for FV.

### Developmental Dysgraphia

Starting with Hinshelwood (1917), many researchers have drawn parallels between the difficulties of those children with specific impairments in reading and writing, and brain-injured adult dyslexics. The same cognitive neuropsychological approach has been applied to developmental cases of dyslexia and dysgraphia with regard to classification, terminology, and etiology for both case (e.g., Temple and Marshall, 1983; Temple, 1985), and group (e.g., Angelelli et al., 2004) studies.

Similar to acquired dyslexia, most children with reading problems also struggle with spelling, as these aspects of literacy share cognitive components and are usually well correlated (e.g., Frith, 1980; Fletcher-Flinn et al., 2004). The difficulties of both are also attributed to the same cognitive impairment (e.g., Shankweiler et al., 1996; Angelelli et al., 2004; Caravolas et al., 2005). For example, Angelelli et al. (2004) described the writing characteristics of 18 Italian dyslexic children, ranging in age from 10 years 8 months (10:8) to 13:1. They reported that they were very slow readers compared with age-matched controls. Their writing errors, mainly on ‘unpredictable’ words, mirrored their reading errors, occurring for the most part on irregular words. They concluded that the children suffered from surface dysgraphia, and their errors were attributed to impairments in lexical access resulting in an overreliance on grapheme–phoneme rules.

Despite the similarities between acquired forms of dyslexia and dysgraphia, and their developmental counterparts, strong claims are problematic (Ellis, 1993). The developmental cases lack known brain trauma, and have developing reading systems that may be inefficient, but are not totally incapable in the way that the acquired ones are. Dyslexic children are generally able to read and write both familiar and unfamiliar words, but to a lesser degree than those making normal progress. Their difficulties are situated in the *learning* of these literacy skills and the establishment of a reading system, rather than a disruption to a well-functioning one. Although the cognitive neuropsychological approach has provided some valuable insights into dyslexia and dysgraphia, a developmental framework is better suited to understanding deficits in learning to read and spell.

### A Developmental Approach

Maul and Ehri (1991) claimed that it was primarily through reading that spellings of words were learnt. The exposure to the correct spellings of words was considered essential due to the vagaries of English spellings making them difficult to predict. Through reading children retained word-specific information in orthographic (lexical) memory that could be accessed for spelling.

Word representations take time to develop, as they require print experience. According to Knowledge Sources theory (Thompson and Fletcher-Flinn, 1993, pp. 32–33), word representations are formed through a learning sequence in which the letters and letter order of words become increasingly complete as new representations are added, and further discrimination is required. As the letters in words become represented, alignments are made between their positional coding, and the temporal coding of the matching phonological component. These alignments are called induced sublexical relations (ISRs), and are used to generate reading responses to new words. They consist of small grapheme–phoneme representations, as well as larger context-dependent sequences of grapheme and corresponding phonological units (Thompson et al., 1996; Thompson, 1999; Thompson and Fletcher-Flinn, 2006). For example, from the orthographic representations of *bat*, *sit*, *cat*, *went*, *get*, the child would induce non-conciously that the final orthographic component –t relates to /–t/.

When the orthographic representations provide sufficient exemplars, more complex ISR associations are formed, such that the ISR for the grapheme *y* in final position of words like *baby* and *happy* is distinguished by position from the *y* in *yes* and *you*, where it has a different sound, and contextually from *by* or *my*, which has a different sound again. Some ISRs take account of the influence of sequences of graphemes and associated phonemes on the mapping of the preceding vowel (e.g., *hold* versus *hot*), or following vowel (*wand* versus *hand*). As more representations are added, so will the number of ISRs expand, creating a system of interlinked representations. The product of this process, ISR knowledge, although mainly implicit, can occasionally become explicit and conscious for some relationships if attention is drawn to them (Thompson, 1999; McKay et al., 2004; Thompson and Fletcher-Flinn, 2006).

Although good readers and spellers are characterized by fast and accurate recall from representations in the orthographic lexicon, it is not necessarily the case that full representation is required for a word to be recalled. In spelling, a detailed representation is far more important for accuracy. This partially explains the common finding in developmental studies of beginning readers that spelling lags behind reading (for a review, Fletcher-Flinn et al., 2004), and may be considerably underdeveloped as shown by a very precocious 3-year-old reader (Fletcher-Flinn and Thompson, 2000). If a word spelling is not fully represented than the missing part must be filled-in from other knowledge sources. According to Ehri (1986), this can be achieved through applying knowledge of the orthographic system, including producing a phonetic spelling, or using a morphographic or analogy strategy. Although orthographic knowledge is mostly implicit, these procedures for filling-in would presumably involve some degree of explicit knowledge gained through instruction, e.g., learning letter–sound relationships.

If reading is critical for spelling, then some form of knowledge transfer must occur from these implicitly formed word, and ISR representations in reading to be available for spelling. Only a few studies have directly examined learning and transfer in developmental dysgraphia. Maul and Ehri (1991) compared the performance of 34 pairs of dysgraphic adolescents (*M* 14 years) with a reading comprehension matched group making normal progress. Fifteen words thought to be unfamiliar in meaning and spelling, were chosen as targets. They found positive transfer for the dysgraphic adolescents to the *same extent* as the comparison group in both an isolated word condition in which the target words were read aloud but not corrected, and in the silent reading of continuous text. It was concluded that their source of difficulty was in ‘remembering’ whole word spellings, which would result in the filling-in of the missing letters by ‘ear.’ Based on their poorer performance on a non-word reading test, and the pronunciations of target words in the isolated reading condition, it was claimed that the dysgraphics had weaker phonological recoding skill with which to do this, and they did not have the knowledge of spelling patterns, and morphophonemic rules to the same extent as the normal spellers with which to generate better guesses.

In another study, Neser (2002, Unpublished) trained seven adolescents (*M* 13:10 years) with specific spelling disability on 40 words differing in regularity (20 high and 20 low). The adolescents were reading-level matched with seven normal-progress adolescents (*M* 12:2). To ensure that the adolescents were not already familiar with the training words, a reading and spelling pretest was given of the entire word pool consisting of 141 matched training and control word pairs. Words that were read incorrectly, as well those that were spelt correctly were eliminated from the word pool along with the matched control words for each participant. The adolescents read the 40 training words in different sentences, with a total of four exposures for each. The normal-progress spellers showed significant gains in spelling, relative to the matching untrained control words, but only on the highly regular words. The dysgraphics showed no gains.

It was suggested that the gains for the normal spellers were facilitated by the activation of existing stored ISRs comprising phoneme–grapheme correspondences constrained by regularity patterns. The small number of reading exposures may have been insufficient for learning new phonological recoding relations for the less regular words. As six of the seven adolescents who were spelling disabled had difficulty learning to read, they may not have had sufficient reading exposure to develop the associative sublexical networks to facilitate transfer from reading to spelling for even the highly regular words. In terms of a more general transfer, as ISRs are position sensitive, the specificity of the grapheme positions and sequences of graphemes of the training words did not feature to the same extent in the control words, and this specificity was suggested as contributing to the non-significant gains on these words for both groups.

The two training studies contrast in outcome, and this is most likely due to methodological differences. In the Maul and Ehri (1991) study, the spelling posttest was given immediately after the treatments, whereas there was a 3-day delay in the Neser (2002, Unpublished) study. The number of participants, training words, amount of exposures, use of untrained control words, and checks on prior knowledge of target word spellings also differed between studies. Nonetheless, their conclusions are similar, and align well with that of JAS, pointing to difficulty in *establishing* orthographic (word) representations from their reading experience, and/or sublexical components (ISRs) from them to facilitate transfer from reading to spelling. Poor visual memory was considered causally related to the difficulties JAS experienced with spelling, but it was not directly tested in the dysgraphic adolescents. However, those in the Maul and Ehri (1991) study had good recall of double letters, which indicated access to some visual information because they could not be guessed at phonetically. The conclusions from these studies are in contrast to a view of developmental dysgraphia as difficulty with *access* to the representations.

The purpose of this study was to examine the cognitive performance, visual memory, and the reading and spelling of an adolescent referred for assessment due to poor spelling. BT, the case reported in this study was in a state integrated secondary school, and similar to JAS had normal reading attainment but further specific testing revealed some deficits. His performance was compared to normal adult readers, and a sample of 12-year-old normal progress readers. These reading level matches were used to examine the extent to which his reading skills and processing differed from them. The intention was to discuss BT’s reading and spelling from a developmental, rather than a cognitive neuropsychological point of view and to draw from it broader implications for theories of developmental dysgraphia with regard to access (Angelelli et al., 2004), or the establishment of orthographic representations (Goulandris and Snowling, 1991; Maul and Ehri, 1991).

## Materials and Methods

### Case History

BT was 15 years, 3 months (15:3) when testing began. He was in Year 10 at a state-integrated high school for boys. As reported by his mother, BT had a normal delivery at full term, and he reached all of his developmental milestones on time, except for his language. He was a late talker and there were concerns about his hearing. From about 8 months, he suffered from ear infections, and grommets were inserted at 3 years.

At 4½ years, he entered school in England, and the teacher reported that he seemed ‘distracted,’ and didn’t always respond to his name. He made poor progress in reading with phonics instruction, which was supported at home with Jolly Phonics. At 6.5 years, he completed the National Curriculum assessments, and scored above average for Mathematics and Science, but below the grade norm for English. When BT was 7 years, the family moved to New Zealand. According to his mother, reading and writing seemed to get worse at first, but by 10 years, he was reading independently and avidly. It is not surprising that BT had initial difficulty as the New Zealand curriculum for reading is based on a text-centered approach, which is supported through books with increasing levels of difficulty (see, Thompson, 1993). This approach would have been different to the phonics instruction that he had been receiving in England.

It was noted that BT’s father had similar difficulty with spelling, and his paternal grandfather was characterized as an “underachiever” despite a high IQ. At school, BT struggled with some ball sports, but he was able to enjoy rugby, swimming, and cross-country, and continues to be involved with a fencing club. He is a good runner and loves hiking and being outdoors. Art is a challenge, although when he was nine, an inspired art teacher encouraged him to use patterns of dabs of paint to imitate Australian Aboriginal paintings. His painting was considered so good that it was framed by the school and still hangs in the Principal’s office. However, like his father, he still finds drawing a problem, and his handwriting is hardly legible. Of the 10 Topic tests for Year 9, BT only managed to finish half of them.

### General Procedure

BT was administered a series of cognitive and literacy tasks over a period of about 1 month, with two to three 1 h sessions per week. The cognitive tasks were administered during the first week, followed in the second week by the standard reading and spelling tasks, and in the last 2 weeks, the experimental reading and spelling tasks. Published data from 12-year-olds (Fletcher-Flinn and Thompson, 2004) and two adult samples (Thompson et al., 2009), as well as the Coltheart and Leahy (1992) samples were used as comparison groups for the various experimental tasks where appropriate. The 12-year-old sample was justified as BT’s reading age on the WRAT4 was shown to be at that level. Presentation was similar to that used for the comparison groups for all of the experimental tasks.

## Assessments

### Cognitive

BT was assessed with the Wechsler Intelligence Scale for Children–Third Edition, Australian Version (WISC–3, Wechsler, 1992). His Verbal IQ was 127, Performance IQ was 113, and his Full Scale IQ was 123. His Index Scores were: Verbal Comprehension 123, Perceptual Organization 126, Freedom from Distraction 129, and Perceptual Speed 83. He achieved a standard score (SS) of 11 on Digit Span forward, and 9 on Digit Span backward, putting him in the Average range for auditory sequential memory. His receptive vocabulary of 110 on the British Picture Vocabulary Scale (BPVS, Dunn et al., 1997) was consistent with his expressive vocabulary on the WISC–3 (SS 12).

All of BT’s subtest scores on the Verbal and Performance Scales of the WISC–3 were in the very superior to above average range for his age. There was a significant disparity between Perceptual Speed and the rest of the Index scores. The combined score put him in the Low Average range. Perceptual Speed is measured by performance on two subtests, Symbol Search and Coding. In Symbol Search a decision must be made about whether target symbols appear in a row of symbols, whereas for Coding, a digit-symbol code must be transcribed as quickly as possible. Both measures involve skills in quick scanning and visual sequential short-term memory, and speed of processing (psychomotor speed). BT performed at an average level on Symbol Search with a SS of 10, but showed a severe deficit on the other subtest (SS 3).

The difference in the subtests is their content, with Symbol Search having figural content, and the Coding subtests having symbolic content. The latter requires a strategy of associative learning, forming paired associations between numbers and shapes. It is sensitive to the ability to learn these associations, and performance can be influenced by a weakness in short-term visual memory of the learned associations. BT made no errors, and it was noted that he performed this task by giving names to the shapes. His poor performance was due to slow processing.

He was also administered the Mazes supplementary subtest from the WISC–3 to further examine his fine motor skills, ability to manipulate a pencil, and planning ability. On this subtest, scores are given for speed and accuracy. He made no errors, and his speed was well within the maximum, completing the two most difficult mazes having time limits of 150 s, in 38 and 31 s. He achieved the maximum SS of 17.

Considering BT’s overall cognitive performance was in the superior range, his performance on the Coding subtest was markedly discrepant, and his verbal mediation strategy was consistent with a visual memory deficit and warranted additional examination.

### Visual Short-Term Memory

Visual sequential memory was assessed with the Illinois Test of Psycholinguistic Abilities Revised (ITPA-R, Kirk et al., 1968). It consists of a set of 17 tiles with arbitrary visual symbols that must be arranged in the sequence shown in the test booklet. For each item, a sequence is shown for 5 s then removed and the participant is asked to place the tiles in the same order. Two points are given for correct items on the first trial, one point if correct on the second trial, and none if both trials are failed. Testing is discontinued after two consecutive items are failed. BT scored in the Average range for his age (Heriot and Beale, 1996), and similar to the Coding subtest on the WISC–3, this score was achieved using an oral strategy.

The Recall of Designs subtest from the British Ability Scales (BAS, Elliott et al., 1978) was administered to BT. In this subtest, 19 geometric designs have to be drawn from memory following a 5 s study period. Points are given for accuracy and ranges from 2 to 0. BT achieved a raw score of 23/38, which is in the Average range for his age (Centile Score 59).

Both visual memory tests are consistent in placing BT in the average range. BT’s performance on visual sequential memory is similar to his score on the WISC–3 Digit Span subtest which measures auditory sequential memory. Although his oral strategy on the sequential memory tests is suggestive of a visual memory deficit, it would have been difficult to code verbally for the recall of geometric designs, and he was not observed doing so. Given his normal scores on the tests requiring visual analysis and visual memory, it does not appear that BT suffers from a visual memory deficit.

### Standardized Tests of Reading and Spelling

The Wide Range Achievement Test 4 (WRAT–4, Wilkinson and Robertson, 2006) was used to test BT’s word reading and spelling skills. For word reading (Combined Form), his SS was in the Average range at 95. This is equivalent to a reading age of approximately 12½. Although it is an acceptable score in the normal range for his age, it is inconsistent with his Superior verbal ability. On the spelling subtest his SS was 82. This is equivalent to an age score of approximately 9–9½. His spelling skills were in the Low Average range, and almost undecipherable. This is in contrast to his excellent fine motor skills as shown on the Mazes subtest of the WISC–3, and his drawing of geometric designs on the BAS subtest. It is possible that his poor handwriting is task dependent. Deuel (1995) refers to this as a ‘material-specific dyspraxia’.

These standardized scores on reading and spelling correspond well to attainment on school-based assessments. BT’s reading comprehension on the Assessment for Teaching and Learning Test (e-Asttle, Ministry of Education, 2005) was one level higher than the average student. On the Progressive Achievement Test Listening Comprehension (PAT, New Zealand Council for Educational Research, 2010), the participant listens to text read by the teacher, and responds to comprehension questions. BT was average (Stanine 6) on this test for his year level. On the Schonell Spelling Test (Schonell, 1932) his spelling age was 9:1, which is similar to his age level performance on the WRAT–4.

## Experimental Tasks: Reading

### Phoneme and Grapho-Phonemic Awareness

The extended Scarborough task (Thompson et al., 2009, Table 2) was used for assessment and comparisons were made with two adult samples, one from New Zealand who had not received phonics instruction in childhood, and the other from Scotland who had received such instruction. In the phoneme awareness task 30 words were presented in aural form and the task was to count the number of the “smallest sounds” in each word, (e.g., four for *socks*). The same words from this task were used in a grapho-phonemic task in which the words were presented in print form. In this task, the participant must read the word, note the number of sounds in the word (awareness score), and underline the letter or letter sequence belonging to each sound (identity score).

BT’s accuracy on the test of phoneme awareness at 60% was close to the adult sample of university students (61%) without phonics instruction. At 77%, he was 0.8 *SD* better than the New Zealand sample for grapho-phonemic awareness (NZ adults, 61%), and 1.85 *SD* better at 63% on grapho-phonemic segmentation (NZ adults, 39%). Neither of these differences were significant at the 0.05 level. His scores were all within 1 *SD* of the adult sample from Scotland who similar to BT had received phonics instruction in childhood. These scores indicated that his performance was at an acceptable adult level.

### Letter Names and Sounds

The same three tasks as in Thompson et al. (2009, Table 2) for giving letter names, letter sounds, and digraph sounds were administered to BT on a computer with speeded instruction. Comparisons were made with the two adult samples. The first two sets comprised the 26 lower case alphabet letters, and the last consisted of 29 digraphs, all presented in random order, in the same session. BT was 100% accurate on the letter name task. On providing phonic sounds to letters, he achieved 62% accuracy, which was within 1 *SD* of the NZ adult sample (75%), and on digraphs at 61% accuracy, he was within 1.2 *SD* of the NZ adult sample (73%). He was significantly lower, *p* < 0.05, than the adult sample with phonics instruction on both giving the phonics sounds for letters (87%), and digraphs (78%) by 2.13 *SD*, and 2.78 *SD*, respectively. BT’s knowledge of phonic sounds for letter names, sounds and digraphs was at acceptable adult levels considering the teaching instruction that he had received since he arrived in New Zealand.

### Reading Non-Words

BT was administered the Coltheart and Leahy (1992, Task 2) non-word task, which consisted of three sets of 20 items, Regular, body-consistent; Regular, body-inconsistent; and Irregular, body-consistent non-words presented in a randomized sequence. There was only one correct regular response for the Regular Consistent non-words, (e.g., *dack*, *ving*), and one correct irregular response for the Irregular Consistent non-words (e.g., *jook* with –*ook* pronounced as in “book”; *vind* with –*ind* pronounced as in “kind”). Regular responses (e.g., *jook* with –*ook* pronounced as in “spook”; *vind* with –*ind* pronounced as in “wind”) were considered less correct, regularization errors. The Inconsistent non-words were scored as correct for either a regular or irregular pronunciation (e.g., *nush* with –*ush* pronounced as in “rush” or “push”). The task was administered on a computer with speeded instruction. Comparisons were made with the 12-year-old NZ sample, the two adult samples (Thompson et al., 2009), and the Coltheart and Leahy samples.

Overall, for the categories of correct *regular* responses to the Regular Consistent and Inconsistent non-words, BT’s combined accuracy was 67% (**Table 1**). He was within 1 *SD* of the 12-year-olds who achieved 70% (*SD* 11%), but less than the Coltheart and Leahy adults (Coltheart and Leahy, 1992, Table 3), and the NZ adults, who averaged 93 and 92% (*SD* 4), respectively.

**Table 1:** Mean percentage of regular and irregular pronunciations for the Coltheart and Leahy non-words varying in regularity and consistency of body spelling for BT and normal-progress and adult readers.

	Regular consistent	Inconsistent	Irregular consistent
**Regular pronunciations**			
BT	55	79	47
NZ 12-year-olds	82 (10.5)	57 (11.2)	33 (14.9)
NZ Adults	92 (4)	84 (7)	10 (9)
**Irregular pronunciations**			
BT	–^a^	11	26
NZ 12-year-olds	–	23 (10.5)	46 (13.9)
NZ adults	–	10 (6)	63 (17)

*^a^Dashes indicate that regular pronunciations do not exist for regular consistent non-words*.

There is one interesting anomaly, BT performed poorly on the Regular Consistent non-words compared to the other samples. He was 2.57 *SD* below the 12-year-olds, which is a significant difference, *p* < 0.05. Of his nine errors, four were pronounced as real words (e.g., *rell* as “real”; *prile* as “pride”), and the other five responses were non-words (e.g., *drace* as “drance”; *hane* as “han”). For the most part, the errors consisted of consonant substitutions, or vowel/consonant additions (each 38%), and vowel digraph/consonant blend reductions (25%). One response seemed to be a combination of errors (“deal” for *stell*). A major source of errors for the children in the Coltheart and Leahy (1992, p. 727) study for Grades 1–3 was the failure to lengthen the vowel for final *e* non-words (between 25 and 30%). Of the eight non-words of this type, BT made 6 errors (75%) errors. However, there were also 6 final *e* non-words of a similar type in the Inconsistent set, and BT pronounced all of these as correct regular responses.

The pattern of accuracy results for BT on the Irregular Consistent non-words was most similar to the adults in the Coltheart and Leahy (1992) study. Unlike the NZ adults and 12-year-olds, his responses to the Irregular Consistent non-words were dominated by regularizations (45%), as were the Coltheart and Leahy adults (49%). The number of correct responses to the Irregular Consistent non-words (25%) was also similar to that sample of adults (28%). As suggested (Fletcher-Flinn and Thompson, 2004, 2007; Fletcher-Flinn, 2014), the large number of regularizations may be a long-term result of early exposure to phonics instruction.

BT’s response times were analyzed for those categories above mean acceptable response rates of 33% or higher (**Table 2**). These included only two categories, the regular responses for Regular Consistent (*Mean* RT = 1018 ms) and Inconsistent (*Mean* RT = 926 ms, *SD* = 226) non-words. There were no outliers (±3 *SD*s), and a repeated measures analysis of variance (ANOVA) by items (e.g., Fletcher-Flinn and Thompson, 2000, 2004, 2007; Beland and Mimouni, 2001; Upton et al., 2003; Fletcher-Flinn, 2014) showed no difference in mean RTs, *F* > 1.

**Table 2:** Mean RTs (ms) of regular and irregular pronunciations for the Coltheart and Leahy non-words varying in regularity and consistency of body spelling for BT and normal-progress readers

	Regular consistent	Inconsistent	Irregular consistent
**Regular pronunciations**			
BT	1018 (228)	926 (224)	1274 (392)
NZ 12-year-olds	1160 (375)	1064 (284)	1279 (623)
**Irregular pronunciations**			
BT	–^a^	1019 (115)	1073 (285)
NZ 12-yearolds	–^a^	1111 (260)	1229 (466)

*^a^Dashes indicate that regular pronunciations do not exist for regular consistent non-words*.

As response times were available for half of the 12-year-olds, BT’s response time averaged over these two categories of regular responses (*Mean* RT 972 ms, *SD* 226) was not significantly different compared to this subsample (1220 ms, *SD* 499). However, it was 2.5 *SD* slower (*p* < 0.05) than the New Zealand adults who averaged 673 ms (*SD* = 138).

Overall, BT’s accuracy and speed for reading non-words was consistent with his reading age, and comparable to the comparison group of 12-year-olds. The one exception was his lower score for Regular Consistent non-words, which was significantly below the comparison group.

### Exemplar Word Reading

BT was administered the Coltheart and Leahy (1992), Tasks 3 and 4 on a computer with speeded instructions. These two tasks consisted of four sets of 20 exemplar words (e.g., back, rush, bush, walk) that contained the rimes from the non-words, and a set of 20 fillers. BT’s mean accuracy was 95%. This was not significantly better (1.4 *SD*) than the full sample of 12-year-olds at 80% (*SD* 11). There was no difference for BT in response times (repeated measures by items ANOVA) across these categories, *F*(3,36) = 0.40, *p* < 1, and his average RT was 702 ms. (*SD* 94 ms). His word reading was significantly faster (2.9 *SD, p* < 0.05) than his non-word reading, which is a normal finding (Ellis, 1993).

## Experimental Tasks: Spelling

### Knowledge of Spelling Errors

According to Ehri (1986, p. 126), after a spelling is written, an assessment can be made as to whether the word “looks right” and has no errors. This validation process involves comparing the spelling against any stored “visual alphabetic information.” To ascertain if BT was visually aware of his errors, the two versions of BT’s typed responses on the WRAT–4 spelling test were printed, and BT was asked to tick the words that were correct. On the Green Form, BT made 22 errors before he achieved the cut-off point of 10 consecutive errors, and on the Blue form he made 12 errors before reaching the cut-off point. Of the 22 errors on the Green form, BT thought five were correctly spelt words, and of the 12 errors on the Blue form, he thought one item (i.e., belive) was correct. It appeared that BT was to a large extent aware of his errors.

### Typing Words on Computer

To explore the possibility that BT’s poor handwriting might have contributed to errors in letter formation (Deuel, 1995), or being unrecognizable, penalized in the scoring, the spelling subtest of the WRAT–4 was repeated. The automatic spell checker was turned off but the procedure was exactly the same as for the handwritten version of the test. The total number of spelling errors on the written form (Blue and Green forms combined) was 26, and on the computer it was 25. The correlation between the hand written and computer version of the test was significant for the Blue form, *r*(23) = 0.65, *p* = 0.001, and likewise for the Green form, *r*(31) = 0.81, *p* = 0.0001. These results show that BT’s errors were fairly consistent across modalities, and there was no advantage in using the computer. This is compatible with a study by Ouellette and Tims (2014) showing no modality effect on orthographic learning. Any claimed advantage for computer use is probably due to a working spell checker.

### Error Analysis: Spelling

An error analysis was carried out on the computer version of the WRAT–4 spelling subtest to explore the types of errors that were made. Of BT’s total number of errors on both forms, 51% were mainly attempts to spell words phonetically. On the Green form, 12 of the 22 were attempts to spell the words phonetically (e.g., *explain* as explane; *kitchen* as kichin; *museum* as mussium). This strategy of spelling also occurred on the Blue Form. Six of the 12 items were phonetic attempts at spelling (e.g., *correct* as cerrect; *ruin* as ruein; *believe* as belive).

All of the errors bore some relationship to the presented word and could be classified as letter substitutions (e.g., c*o*rrect as c*e*rrect), additions (e.g, brief as brief*e*), deletions (e.g., mater*i*al as materal), or a combination of these errors (e.g., c*i*r*c*l*e* as c*u*r*a*l). There were no transpositions. The largest number of errors was of the mixed type (59%) and occurred on words of more than one syllable. Of these, 38% were due to a letter(s) deletion resulting in the missing of a full syllable, in addition to another error(s) (e.g., rea*son*able as resnabal; ini*t*i*ati*ve as inishive). A noticeable consistent error was the lack of placing the letter u after q (i.e., e*qu*ipment as e*q*ment; lo*qu*acious as low*q*ashus; *qu*antity as *q*wantity). BT also showed consistency in using the suffix –*al* at the end of words (i.e., circle as cur*al*; material as mater*al*; reasonable as resnab*al*; imperturbable as inperturb*al*). Other than the misspelling of *material*, –*al* was used as a substitute for –*le* (as in cur*a*l for circle). These consistent errors across words indicate that BT lacked significant knowledge of standard orthographic conventions for spelling.

## General Discussion

BT appears to be a compensated dyslexic with residual problems in spelling. Both his visual memory for designs, and his visual and auditory sequential memory were normal for his age, and he was visually aware of his spelling errors. He performed at an acceptable level on a standard test of isolated word reading, as well as on school-based measures of text reading, and his accuracy for reading single syllable words was age appropriate. His letter–sound skills and phoneme awareness were consistent with adult levels. Although BT’s spelling was in the low average range, his scores on the two standardized spelling tests equated to an age score of approximately 9–9½ years, far below what would be expected given his IQ and reading age. Most of his errors were phonological attempts to spell and occurred on words of more than one syllable. There were some consistent errors, with the most unexpected being the lack of placing a *u* following the letter *q*, which is a highly predictable orthographic regularity in English.

Despite his normal standardized scores in reading, BT showed some subtle deficits. His accuracy and speed of processing non-words was less than expected for his chronological age but consistent with his reading age. His pattern of responses for Irregular Consistent non-words was similar to other samples with such instruction, showing a strong regularization effect on pronunciation (Coltheart and Leahy, 1992; Fletcher-Flinn and Thompson, 2007; Thompson et al., 2009). This preponderance of regular responses on these non-words having highly consistent irregular rimes indicates the dominant use of grapheme–phoneme units for processing, showing the long-term effects of early instruction in phonics.

Considering his more than adequate knowledge of letter–sound correspondences, and his phonological processing bias, BT’s poor performance on Regular Consistent non-words was surprising. His accuracy was significantly less than a sample of normal-progress 12-year-olds. BT’s error responses consisted of almost an equal number of real words and incorrect non-words, resembling the performance of much younger children. It is not immediately clear why BT made so many errors on this set of non-words, with 67% occurring on non-words necessitating the elongation of the vowel (rule of *e*). In contrast, he had no trouble with this type of non-word for the Inconsistent set, making no errors. The only notable difference between the set of final *e* non-words were the medial vowels. The Regular Consistent set were comprised of an equal number of the medial vowels *a* and *i* (e.g., *yane*, *prile*), whereas the Inconsistent set had medial vowel components of only *o* (e.g., *brone*). These errors indicate a reading processing system inefficient at forming context-dependent ISRs that consist of position sensitive sequences of graphemes and matching phonology, including the rime.

Although not directly tested, but based on BT’s consistent spelling errors that failed to take advantage of orthographic regularities, it seems reasonable to suggest that transfer from reading to spelling was limited. It is likely that this is due to his earlier reading difficulty, which for a significant period of time would have constrained the size of the orthographic lexicon due to insufficient print exposure. There is some evidence that processing within a reading system reaches stability early, as shown by a very precocious reader (Fletcher-Flinn, 2014). If so, it may be that in BT’s case, the reading processing system remained biased toward the formation of smaller ISR units, consisting of simple grapheme–phoneme relationships. This might have been exacerbated by early school instruction in phonics, which draws attention to individual context-free letters and sounds. The consequence for spelling is the generation of a phonetic (sound–letter) spelling to fill the gaps for partially represented words, which would result in many inaccuracies.

To summarize, BT has no difficulty with *access* to his orthographic lexicon in that he can read words that he knows both quickly and accurately. This probably characterizes most developmental cases. It is when BT has to *generate* a reading response for unfamiliar words (non-words) that he is slower, and far less accurate. This is consistent with his spelling performance. He can spell words that he can access, as presumably they are completely represented, but he has difficulty with those that are not, in which case the missing letters must be provided. In BT’s case, reading and spelling are limited by small unit (ISR) activation and processing, which is adequate for reading to a large extent, but does not work as well for spelling. The difficulty experienced may not be in establishing orthographic (word) representations due to poor visual memory as this was in the normal range for BT. It would seem that the primary difficulty is in the *formation* of context-dependent ISRs that include the rime, and other regularities from the orthographic representations. These, in a reciprocal manner, would support the further establishment of orthographic representations, and through interconnected activation facilitate word spellings. Some ISR knowledge might also become available during the conscious process of reflecting on incomplete word spellings, and this information could also contribute to the generation of the missing letters.

This case study raises issues with regard to contemporary theories of developmental dysgraphia. Cognitive neuropsychological explanations based on dual route theory (Coltheart, 2005) have provided valuable insights, but learning and the establishment of a reading system are not considered. Viewed from a developmental perspective, *learning* to read and spell is an overriding consideration. However, the picture appears far more complex than is implied by propositions pertaining to lack of access (Angelelli et al., 2004), or the failure to establish (complete) orthographic representations due to poor visual memory (Goulandris and Snowling, 1991), or ‘remembering’ the words (Maul and Ehri, 1991). The reading system comprises sublexical representations (ISRs) that have a substantial role in learning to read, and by the activation of associative networks, facilitate transfer from reading to spelling. These ISRs are formed from orthographic (word) representations and their matching phonological components. If there is difficulty in learning to read then both the orthographic representations and the resulting ISRs will be limited, resulting in delay in both aspects of literacy relative to those making normal progress.

In the case of BT, by adolescence his reading proficiency was in the normal range but his spelling had not caught up to the same extent. This asynchrony is similar to normal progress beginning readers. Small position-sensitive ISRs are formed initially and these would, to a large extent, typify those available in the reading system of beginners, and similarly, BT. What is lacking in both are complex ISRs that include the rime and other orthographic regularities. For beginning readers this is due to insufficient orthographic representations, as reading experience is limited. BT, on the other hand, has sufficient reading experience but his system of ISRs has remained limited. Whether this is due to the early stability of the reading system biasing ISR formation toward smaller units as suggested, remains an open question, and warrants further research with longitudinal case studies, and with larger samples of developmental dysgraphics. As reading is essential for spelling, then more studies carefully documenting the reading processes of dysgraphics, and involving experimental tests of transfer are needed.

Although a thorough review of educational support strategies is beyond the scope of this paper, there are some implications from this research that might be incorporated into current programs. Considerable practice in reading should be encouraged, as it is through reading that orthographic knowledge for spellings are learnt. As this knowledge is mainly implicit, it might be useful to point out orthographic regularities and patterns in words from learners’ reading vocabulary (McKay et al., 2004). Management could include the use of word processors, and untimed tests (Deuel, 1995). This ‘bypass’ method would make up for some of the adverse effects of poor spelling and slow writing (as shown by BT’s school test results) due to the mental effort required to generate correct spellings.

## Author Contributions

The author confirms being the sole contributor of this work and approved it for publication.

## Conflict of Interest Statement

The author declares that the research was conducted in the absence of any commercial or financial relationships that could be construed as a potential conflict of interest.

## References

[B1] Adi-Japha E. Landau Y. E. Frenkel L. Teicher M. Gross-Tsur V. (2007). ADHD and dysgraphia. *Cortex* 43 700–709. 10.1016/S0010-9452(08)70499-4 17710822

[B2] Angelelli P. Judica A. Spinelli D. Zoccolotti P. Luzzatti C. (2004). Characteristics of writing disorders in Italian dyslexic children. *Cogn. Behav. Neurol.* 17 18–31. 10.1097/00146965-200403000-00003 15209222

[B3] Beland R. Mimouni Z. (2001). Deep dyslexia in the two languages of an Arabic/French bilingual patient. *Cognition* 82 77–126. 10.1016/S0010-0277(01)00148-2 11716831

[B4] Bruck M. (1990). Word recognition skills of adults with childhood diagnoses of dyslexia. *Dev. Psychol.* 26 439–454. 10.1037/0012-1649.26.3.439

[B5] Caravolas M. Volin J. Hulme C. (2005). Phoneme awareness is a key component of alphabetic literacy skills in consistent and inconsistent orthographies: evidence from Czech and English children. *J. Exp. Child Psychol.* 92 107–139. 10.1016/j.jecp.2005.04.003 16038927

[B6] Coltheart M. (2005). “Modeling reading: the dual route approach,” in *The Science of Reading: a Handbook*, eds Snowling M. J. Hulme C. (Malden, MA: Blackwell Publisher), 6–23.

[B7] Coltheart V. Leahy J. (1992). Children’s and adults’ reading of non words: Effects of regularity, and consistency. *J. Exp. Psychol. Learn. Mem. Cogn.* 18 718–729. 10.1037/0278-7393.18.4.718 1385612

[B8] Critchley M. Critchley E. R. (1978). *Dyslexia Defined.* London: Heinemann.

[B9] Deuel R. K. (1995). Developmental dysgraphia and motor skills disorders. *J. Child Neurol.* 10 S6–S8. 10.1177/08830738950100S103 7538525

[B10] Dunn L. M. Dunn L. M. Whetton C. Burley J. (1997). *British Picture Vocabulary Scale*, 2nd Edn Windsor: NFER-Nelson.

[B11] Ehri L. C. (1986). Sources of difficulty in learning to spell and read. *Adv. Devel. Behav. Pediat.* 7 121–195.

[B12] Elliott C. D. Murray D. J. Pearson L. S. (1978). *British Ability Scales.* Windsor: National Foundation For Educational Research.

[B13] Ellis A. W. (1993). *Reading, Writing and Dyslexia: A Cognitive Analysis*, 2nd Edn Hove: Lawrence Erlbaum Associates Ltd.

[B14] Fletcher-Flinn C. M. (2014). Learning to read as the formation of a dynamic system: evidence for dynamic stability in phonological recoding. *Front. Psychol.* 5:660 10.3389/fpsyg.2014.00660 PMC4080383 25071635

[B15] Fletcher-Flinn C. M. Shankweiler D. Frost S. J. (2004). Coordination of reading and spelling in early literacy development: an examination of the discrepancy hypothesis. *Read. Writ.* 17 617–644. 10.1023/B:READ.0000044297.85675.f5

[B16] Fletcher-Flinn C. M. Thompson G. B. (2000). Learning to read with underdeveloped phonemic awareness but lexicalized recoding: a case study of a three-year-old. *Cognition* 74 177–208. 10.1016/S0010-0277(99)00072-4 10617781

[B17] Fletcher-Flinn C. M. Thompson G. B. (2004). A mechanism of implicit lexicalized phonological recoding used concurrently with underdeveloped explicit letter sound skills in both precocious and normal reading development. *Cognition* 90 303–335. 10.1016/S0010-0277(03)00162-8 14667699

[B18] Fletcher-Flinn C. M. Thompson G. B. (2007). Dissociation between deficits in explicit procedures and implicit processes in the visual-spatial and the phonological systems during reading acquisition. *Cogn. Neuropsychol.* 24 471–484. 10.1080/02643290701423689 18416502

[B19] Flyvbjerg B. (2006). Five misunderstandings about case-study research. *Qual. Inq.* 12 219–245. 10.1177/1077800405284363

[B20] Frith U. (1980). “Unexpected spelling problems,” in *Cognitive Processes in Spelling*, ed. Frith U. (London: Academic Press), 495–515.

[B21] Goulandris N. K. Snowling M. (1991). Visual memory deficits: A plausible cause of developmental dyslexia? Evidence from a single case study. *Cogn. Neuropsychol.* 8 127–154. 10.1080/02643299108253369

[B22] Heriot S. Beale I. (1996). New zealand norms for the visual sequential memory test of the illinois test of psycholinguistic abilities–revised. *New Zeal. J. Psychol.* 25 1–7.

[B23] Hinshelwood J. (1917). *Congenital Word Blindness.* London: HK Lewis.

[B24] Kirk S. A. McCarthy J. J. Kirk W. D. (1968). *Illinois Test of Psycholinguistic Abilities.* Urbana, IL: University of Illinois Press.

[B25] Maul B. D. K. Ehri L. C. (1991). “Memory for spellings in normal and dysgraphic spellers,” in *Written Language Disorders*, ed. Joshi M. (Amsterdam: Kluwer Academic Publishers), 25–42.

[B26] McKay M. F. Fletcher-Flinn C. M. Thompson G. B. (2004). New theory for understanding reading and reading disability. *Aust. J. Learn. Disabil.* 9 3–7. 10.1080/19404150409546758

[B27] Miceli G. Silveri M. C. Caramazza A. (1985). Cognitive analysis of a case of pure dysgraphia. *Brain Lang.* 25 187–212. 10.1016/0093-934X(85)90080-X 4063789

[B28] Miles T. R. (1983). *Dyslexia: the Pattern of Difficulties.* Oxford: Blackwell.

[B29] Ministry of Education (2005). *Assessment Tools for Teaching and Learning (e-asTTle).* Available at: http://e-asttle.tki.org.nz/

[B30] New Zealand Council for Educational Research (2010). *Progressive Achievement Tests: Listening Comprehension.* Wellington, NZ: NZCER Press.

[B31] Nicolson R. I. Fawcett A. J. (2011). Dyslexia, dysgraphia, procedural learning and the cerebellum. *Cortex* 47 117–127. 10.1016/j.cortex.2009.08.016 19818437

[B32] Ouellette G. Tims T. (2014). The write way to spell: printing vs. typing effects on orthographic learning. *Front. Psychol.* 5:117 10.3389/fpsyg.2014.00117 PMC3923165 24592247

[B33] Schonell F. J. (1932). *Schonell Spelling Test.* Cheltenham. GB: Nelson Thornes Ltd Available at: http://www.academicedge.co.nz/download/schonell-spelling-test.pdf?inline

[B34] Shankweiler D. Lundquist E. Dreyer L. G. Dickinson C. C. (1996). Reading and spelling difficulties in high school students: causes and consequences. *Read. Writ.* 8 267–294. 10.1007/BF00420279

[B35] Snowling M. J. (2013). Early identification and interventions for dyslexia: a contemporary view. *J. Res. Spec. Educ. Needs* 13 7–14. 10.1111/j.1471-3802.2012.01262.x 26290655 PMC4538781

[B36] Temple C. M. (1985). Developmental surface dysgraphia: a case report. *Appl. Psycholing.* 6 391–406. 10.1017/S0142716400006329

[B37] Temple C. M. Marshall J. C. (1983). A case study of developmental phonological dyslexia. *Br. J. Psychol.* 74 517–533. 10.1111/j.2044-8295.1983.tb01883.x 6640236

[B38] Thompson G. B. (1993). “Reading instruction for the initial years in New Zealand schools,” in *Reading Acquisition Processes*, eds Thompson G. B. Tunmer W. E. Nicholson T. (Clevedon: Multilingual Matters), 148–154.

[B39] Thompson G. B. (1999). “The processes of learning to identify words,” in *Learning to Read: Beyond Phonics and Whole Language*, eds Thompson G. B. Nicholson T. (New York, NY: Teachers College Press), 25–54.

[B40] Thompson G. B. Connelly V. Fletcher-Flinn C. M. Hodson S. J. (2009). The nature ofskilled adult reading varies with type of instruction in childhood. *Mem. Cogn.* 37 223–234. 10.3758/MC.37.2.223 19223571

[B41] Thompson G. B. Cottrell D. S. Fletcher-Flinn C. M. (1996). Sublexical orthographic-phonological relations early in the acquisition of reading: the knowledge sources account. *J. Exp. Child Psychol.* 62 190–222. 10.1006/jecp.1996.0028 8812047

[B42] Thompson G. B. Fletcher-Flinn C. M. (1993). “A theory of knowledge sources and procedures for reading acquisition,” in *Reading Acquisition Processes*, eds Thompson G. B. Tunmer W. E. Nicholson T. (Clevedon: Multilingual Matters), 20–73.

[B43] Thompson G. B. Fletcher-Flinn C. M. (2006). “Lexicalized implicit learning in reading acquisition: the knowledge sources theory,” in *Cognition and Language: Perspectives from NewZealand*, eds Fletcher-Flinn C. M. Haberman G. M. (BowenHills, QLD: Australian Academic Press), 141–156.

[B44] Upton N. J. Hodgson T. L. Plant G. T. Wise R. J. S. Leff A. P. (2003). Bottom up and top down effects on reading saccades A case study. *J. Neurol. Neurosurg. Psychiatry* 74 1423–1428. 10.1136/jnnp.74.10.1423 14570838 PMC1757385

[B45] Wechsler D. (1992). *Wechsler Intelligence Scale for Children*, 3rd Edn Sydney: Psychological Corporation.

[B46] Wilkinson G. S. Robertson J. G. (2006). *Wide Range Achievement Test 4.* Florida, FL: Psychological Assessment Resources, Inc.

